# Circulating Mitochondrial DNA in Patients in the ICU as a Marker of Mortality: Derivation and Validation

**DOI:** 10.1371/journal.pmed.1001577

**Published:** 2013-12-31

**Authors:** Kiichi Nakahira, Sun-Young Kyung, Angela J. Rogers, Lee Gazourian, Sojung Youn, Anthony F. Massaro, Carolina Quintana, Juan C. Osorio, Zhaoxi Wang, Yang Zhao, Laurie A. Lawler, Jason D. Christie, Nuala J. Meyer, Finnian R. Mc. Causland, Sushrut S. Waikar, Aaron B. Waxman, Raymond T. Chung, Raphael Bueno, Ivan O. Rosas, Laura E. Fredenburgh, Rebecca M. Baron, David C. Christiani, Gary M. Hunninghake, Augustine M. K. Choi

**Affiliations:** 1 Division of Pulmonary and Critical Care Medicine, Department of Medicine, Brigham and Women's Hospital, Harvard Medical School, Boston, Massachusetts, United States of America; 2 Department of Medicine, Weill Cornell Medical College, New York, New York, United States of America; 3 Department of Internal Medicine, Gachon University Gil Hospital, Incheon, South Korea; 4 Channing Division of Network Medicine, Brigham and Women's Hospital, Harvard Medical School, Boston, Massachusetts, United States of America; 5 Division of Pulmonary and Critical Care Medicine, Department of Medicine, Stanford University, Stanford, California, United States of America; 6 Department of Environmental Health, Harvard School of Public Health, Boston, Massachusetts, United States of America; 7 Division of Pulmonary, Allergy and Critical Care Medicine, Department of Medicine, University of Pennsylvania, Philadelphia, Pennsylvania, United States of America; 8 Renal Division, Department of Medicine, Brigham and Women's Hospital, Boston, Massachusetts, United States of America; 9 Division of Gastroenterology, Department of Medicine, Massachusetts General Hospital, Harvard Medical School, Boston, Massachusetts, United States of America; 10 Division of Thoracic Surgery, Department of Surgery, Brigham and Women's Hospital, Boston, Massachusetts, United States of America; 11 Pulmonary and Critical Care Unit, Massachusetts General Hospital, Harvard Medical School, Boston, Massachusetts, United States of America; 12 Samsung Medical Center, Sungkyunkwan University School of Medicine, Seoul, South Korea; Free University of Brussels, Belgium

## Abstract

**Background:**

Mitochondrial DNA (mtDNA) is a critical activator of inflammation and the innate immune system. However, mtDNA level has not been tested for its role as a biomarker in the intensive care unit (ICU). We hypothesized that circulating cell-free mtDNA levels would be associated with mortality and improve risk prediction in ICU patients.

**Methods and Findings:**

Analyses of mtDNA levels were performed on blood samples obtained from two prospective observational cohort studies of ICU patients (the Brigham and Women's Hospital Registry of Critical Illness [BWH RoCI, *n* = 200] and Molecular Epidemiology of Acute Respiratory Distress Syndrome [ME ARDS, *n* = 243]). mtDNA levels in plasma were assessed by measuring the copy number of the NADH dehydrogenase 1 gene using quantitative real-time PCR. Medical ICU patients with an elevated mtDNA level (≥3,200 copies/µl plasma) had increased odds of dying within 28 d of ICU admission in both the BWH RoCI (odds ratio [OR] 7.5, 95% CI 3.6–15.8, *p* = 1×10^−7^) and ME ARDS (OR 8.4, 95% CI 2.9–24.2, *p* = 9×10^−5^) cohorts, while no evidence for association was noted in non-medical ICU patients. The addition of an elevated mtDNA level improved the net reclassification index (NRI) of 28-d mortality among medical ICU patients when added to clinical models in both the BWH RoCI (NRI 79%, standard error 14%, *p*<1×10^−4^) and ME ARDS (NRI 55%, standard error 20%, *p* = 0.007) cohorts. In the BWH RoCI cohort, those with an elevated mtDNA level had an increased risk of death, even in analyses limited to patients with sepsis or acute respiratory distress syndrome. Study limitations include the lack of data elucidating the concise pathological roles of mtDNA in the patients, and the limited numbers of measurements for some of biomarkers.

**Conclusions:**

Increased mtDNA levels are associated with ICU mortality, and inclusion of mtDNA level improves risk prediction in medical ICU patients. Our data suggest that mtDNA could serve as a viable plasma biomarker in medical ICU patients.

*Please see later in the article for the Editors' Summary*

## Abstract 2

In this paper, Choi and colleagues analyzed levels of mitochondrial DNA in two prospective observational cohort studies and found that increased mtDNA levels are associated with ICU mortality, and improve risk prediction in medical ICU patients. The data suggests that mtDNA could serve as a viable plasma biomarker in MICU patients.

## Editors' Summary

**Background:**

Intensive care units (ICUs, also known as critical care units) are specialist hospital wards that provide care for people with life-threatening injuries and illnesses. In the US alone, more than 5 million people are admitted to ICUs every year. Different types of ICUs treat different types of problems. Medical ICUs treat patients who, for example, have been poisoned or who have a serious infection such as sepsis (blood poisoning) or severe pneumonia (inflammation of the lungs); trauma ICUs treat patients who have sustained a major injury; cardiac ICUs treat patients who have heart problems; and surgical ICUs treat complications arising from operations. Patients admitted to ICUs require constant medical attention and support from a team of specially trained nurses and physicians to prevent organ injury and to keep their bodies functioning. Monitors, intravenous tubes (to supply essential fluids, nutrients, and drugs), breathing machines, catheters (to drain urine), and other equipment also help to keep ICU patients alive.

**Why Was This Study Done?:**

Although many patients admitted to ICUs recover, others do not. ICU specialists use scoring systems (algorithms) based on clinical signs and physiological measurements to predict their patients' likely outcomes. For example, the APACHE II scoring system uses information on heart and breathing rates, temperature, levels of salts in the blood, and other signs and physiological measurements collected during the first 24 hours in the ICU to predict the patient's risk of death. Existing scoring systems are not perfect, however, and “biomarkers” (molecules in bodily fluids that provide information about a disease state) are needed to improve risk prediction for ICU patients. Here, the researchers investigate whether levels of circulating cell-free mitochondrial DNA (mtDNA) are associated with ICU deaths and whether these levels can be used as a biomarker to improve risk prediction in ICU patients. Mitochondria are cellular structures that produce energy. Levels of mtDNA in the plasma (the liquid part of blood) increase in response to trauma and infection. Moreover, mtDNA activates molecular processes that lead to inflammation and organ injury.

**What Did the Researchers Do and Find?:**

The researchers measured mtDNA levels in the plasma of patients enrolled in two prospective observational cohort studies that monitored the outcomes of ICU patients. In the Brigham and Women's Hospital Registry of Critical Illness study, blood was taken from 200 patients within 24 hours of admission into the hospital's medical ICU. In the Molecular Epidemiology of Acute Respiratory Distress Syndrome study (acute respiratory distress syndrome is a life-threatening inflammatory reaction to lung damage or infection), blood was taken from 243 patients within 48 hours of admission into medical and non-medical ICUs at two other US hospitals. Patients admitted to medical ICUs with a raised mtDNA level (3,200 or more copies of a specific mitochondrial gene per microliter of plasma) had a 7- to 8-fold increased risk of dying within 28 days of admission compared to patients with mtDNA levels of less than 3,200 copies/µl plasma. There was no evidence of an association between raised mtDNA levels and death among patients admitted to non-medical ICUs. The addition of an elevated mtDNA level to a clinical model for risk prediction that included the APACHE II score and biomarkers that are already used to predict ICU outcomes improved the net reclassification index (an indicator of the improvement in risk prediction algorithms offered by new biomarkers) of 28-day mortality among medical ICU patients in both studies.

**What Do These Findings Mean?:**

These findings indicate that raised mtDNA plasma levels are associated with death in medical ICUs and show that, among patients in medical ICUs, measurement of mtDNA plasma levels can improve the prediction of the risk of death from the APACHE II scoring system, even when commonly measured biomarkers are taken into account. These findings do not indicate whether circulating cell-free mtDNA increased because of the underlying severity of illness or whether mtDNA actively contributes to the disease process in medical ICU patients. Moreover, they do not provide any evidence that raised mtDNA levels are associated with an increased risk of death among non-medical (mainly surgical) ICU patients. These findings need to be confirmed in additional patients, but given the relative ease and rapidity of mtDNA measurement, the determination of circulating cell-free mtDNA levels could be a valuable addition to the assessment of patients admitted to medical ICUs.

**Additional Information:**

Please access these websites via the online version of this summary at http://dx.doi.org/10.1371/journal.pmed.1001577.

- The UK National Health Service Choices website provides information about intensive care
- The Society of Critical Care Medicine provides information for professionals, families, and patients about all aspects of intensive care
- MedlinePlus provides links to other resources about intensive care (in English and Spanish)
- The UK charity ICUsteps supports patients and their families through recovery from critical illness; its booklet Intensive Care: A Guide for Patients and Families is available in English and ten other languages; its website includes patient experiences and relative experiences of treatment in ICUs
- Wikipedia has a page on ICU scoring systems (note that Wikipedia is a free online encyclopedia that anyone can edit; available in several languages)

## Introduction

Multidimensional scoring systems based on commonly measured clinical and physiologic parameters help to improve the prognostication of patients admitted to intensive care units (ICUs) [1]. Despite the improvement in prognostication there is a need to develop biomarkers that can help clinicians to further improve risk prediction for patients in the ICU [2]. While many biomarkers (>170) have been evaluated in various ICU subpopulations, few have been tested in large populations, or add substantially to the predictive value of multidimensional scoring systems [1]. Only a small number of biomarkers reflecting the severity of critical illness are commonly measured in clinical practice (e.g., lactate and procalcitonin) [3],[4]. Accumulating evidence suggests that cellular mitochondrial DNA (mtDNA) levels are decreased [5],[6] and circulating cell-free mtDNA levels are increased in response to stimuli such as trauma [7] and microbial infection [8]–[10]. Moreover, mtDNA acts as a damage-associated molecular pattern [11] that can drive molecular processes, leading to inflammatory responses and organ injuries [7],[10],[12],[13]. Based on these findings we hypothesized that circulating cell-free mtDNA levels would be associated with mortality and would improve risk prediction in ICU patients. The objective of this study was to test whether measuring circulating cell-free mtDNA is useful as a biomarker in the ICU. To our knowledge this is the first study to provide replicated evidence that plasma mtDNA levels both are associated with mortality and improve risk prediction in the field of critical care illness.

## Methods

The protocols for patient recruitment and data collection for the two patient cohorts, the Brigham and Women's Hospital Registry of Critical Illness (BWH RoCI, primary patient cohort) and the Molecular Epidemiology of Acute Respiratory Distress Syndrome (ME ARDS, replication cohort), have been described previously [14]–[16] and are discussed in detail in Text S1 in Supporting Information S1. In brief, from 24 July 2008 to 14 June 2011, 200 patients were prospectively recruited into the BWH RoCI study from the Brigham and Women's Hospital medical intensive care unit (MICU), for whom clinical phenotype data, including mortality, and blood plasma samples were subsequently collected. Acute kidney injury was defined as a ≥0.5 mg/dl increase in serum creatinine [17]. Presence of respiratory, heart, and liver failure were defined by admitting physician report. For replication, we selected 243 patients from the ME ARDS study, which recruited patients from ICUs at Massachusetts General Hospital from 10 November 2002 to 16 June 2010 and at Beth Israel Deaconess Medical Center from 1 February 2007 to 24 April 2010, for whom both 28-d mortality data and a blood plasma sample had been collected. In addition to baseline demographic data, assessments of sepsis and acute respiratory distress syndrome (ARDS) were performed, and Acute Physiology and Chronic Health Evaluation (APACHE) II scores were measured [18]–[20]. 28-d mortality [21] (in both cohorts) and in-hospital mortality (in the BWH RoCI cohort) were ascertained from patient medical records, and long-term mortality was assessed in the BWH RoCI cohort by linking the cohort to the US Social Security Administration's Death Master File [22]. Written informed consent and institutional review board approval were obtained.

### Preparation and Quantification of Plasma DNA

In the BWH RoCI study, blood samples were drawn and transferred into blood collection tubes within 24 h of study inclusion, and processed within 2 h after venipuncture [14]. In the ME ARDS study, blood samples were drawn and transferred into blood collection tubes within 48 h after the establishment of a diagnosis [23].

#### DNA isolation from plasma

Plasma used in this study (from both RoCI and ME ARDS) was collected in EDTA-coated blood collection tubes. The plasma samples were stored at −80°C. DNA was isolated from plasma using the DNeasy Blood and Tissue Kit (#69504; Qiagen), according to the manufacture's manual [12]. Samples were thawed on ice and were then mixed briefly by vortex. The 100 µl of plasma was mixed with 100 µl of PBS, followed by brief vortex. The diluted plasma was centrifuged at 700*g* at 4°C for 5 min, and the supernatant (190 µl) was carefully saved by avoiding touching any pellets and the bottom of the tubes with pipette tips. The obtained supernatant was further centrifuged at 18,000*g* at 4°C for 15 min, and the resulting supernatant (170 µl) was carefully saved. Contamination of cells, cell debris, or pellets into supernatant might lead to a significant change of the results. The obtained supernatant was processed for DNA isolation. In brief, we incubated plasma samples with lysis buffer (included in the kit) and proteinase K at 56°C for 15 min. At the final step of DNA isolation, DNA was eluted in 200 µl of elution buffer (included in the kit). For the quantitative real-time polymerase chain reaction (qPCR) assay, the DNA solution was further diluted 10 times with nuclease-free deionized, distilled H_2_O.

#### Primers and qPCR

DNA level in diluted samples was measured by SYBR Green dye-based qPCR assay using a PRISM 7300 sequence detection system (Applied Biosystems). The primer sequences were as follows: human NADH dehydrogenase 1 gene (mtDNA): forward 5′-ATACCCATGGCCAACCTCCT-3′, reverse 5′-GGGCCTTTGCGTAGTTGTAT-3′ [7],[24]; human β-globin (nuclear DNA): forward 5′-GTGCACCTGACTCCTGAGGAGA-3′, reverse 5′-CCTTGATACCAACCTGCCCAG-3′ [25]; bacterial 16S ribosomal RNA: forward 5′- CGTCAGCTCGTGTTGTGAAA-3′, reverse 5′-GGCAGTCTCCTTGAGTTCC-3′ [7]. Plasmid DNA with complementary DNA sequences for human mtDNA was obtained from ORIGENE (SC101172), and plasmid DNA with complementary DNA sequences for human nuclear DNA was obtained from Sino Biological. Concentrations were converted to copy number using the formula; mol/gram×molecules/mol = molecules/gram, via a DNA copy number calculator (http://cels.uri.edu/gsc/cndna.html; University of Rhode Island Genomics and Sequencing Center) [26],[27]. DNA solutions were diluted in 10-fold serial dilutions and used as standards.

**Figure 1:**
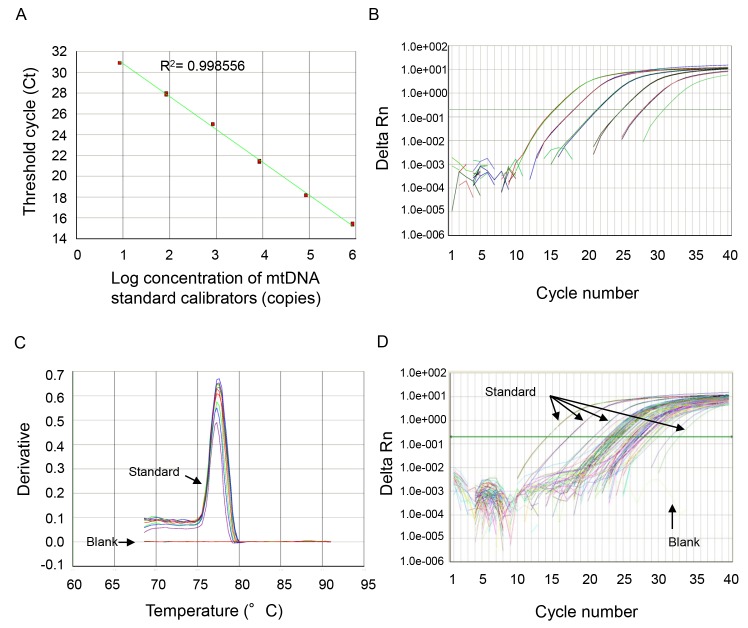
Representative standard curve, amplification plot, and dissociation curve, and amplification plot from the patients' samples. (A) NADH dehydrogenase 1 complementary DNA was serially (1∶10) diluted to prepare a series of calibrators (mtDNA standard) with known DNA copy number. The assay was linear over the range (8.48–848000 copies) of DNA copy numbers (*R* ^2^ = 0.997866). (B) The amplification plot (ΔRn versus cycle [log] view) shows no irregular amplification for the standard diluents (848,000, 84,800, 8,480, 848, 84.8, 8.48). (C) The dissociation curve shows a single melting temperature of the specific products generated with standard template. Also, there is no melting temperature observed in the no-template control wells (blank). These dissociation curves indicate that the reactions are free of primer-dimer or any other spurious products. (D) Similar amplification plots were observed after qPCR using patients' samples. The amplification curves generated from human samples were paralleled with the curves from standards and were in the range of amplification plots for standard diluents.

The thermal profile for detecting mtDNA was carried out as follows: an initiation step for 2 min at 50°C is followed by a first denaturation step for 10 min at 95°C and a further step consisting of 40 cycles for 15 s at 95°C and for 1 min at 60°C. A representative standard curve, dissociation curve, and amplification plot are shown in Figure 1.

All samples were analyzed in duplicate, and a no-template control was included in every analysis. mtDNA levels in all of the plasma analyses were expressed in copies per microliter of plasma based on the following calculation [28]:
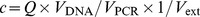
(1)where *c* is the concentration of DNA in plasma (copies/microliter plasma); *Q* is the quantity (copies) of DNA determined by the sequence detector in a PCR; *V*_DNA_ is the total volume of plasma DNA solution obtained after extraction, typically 200 µl per extraction; *V*_PCR_ is the volume of plasma DNA solution used for PCR, typically 5 µl of ten times diluted plasma DNA solution; and *V*_ext_ is the volume of plasma extracted, typically 50–100 µl.

The specificity of mtDNA primers was examined using bacterial DNA (Text S2 in Supporting Information S1; Figure S1) and by BLAST search (Text S3 and Tables S1 and S2 in Supporting Information S1).

### Procalcitonin Measurement

Plasma level of procalcitonin was measured using the Procalcitonin Human ELISA (Enzyme-Linked Immunosorbent Assay) Kit (ab100630; Abcam).

### Statistical Analysis

mtDNA level was assessed as both a continuous and a binary variable, where indicated. Because mtDNA levels were non-normally distributed, continuous levels from the BWH RoCI cohort were assessed after log_10_ transformation and dichotomized using visual inspection and iterative methods (using c-statistics) [29] to select the level of mtDNA that maximized the area under the curve for the prediction of 28-d mortality (mtDNA ≥3,200 copies/µl versus <3,200 copies/µl). This level was then replicated in the ME ARDS cohort. Survival was assessed as both a binary and a time-dependent variable. Bivariate analyses were conducted with Fisher's exact tests (for categorical variables) and Wilcoxon rank-sum tests (for continuous variables). Both bivariate and multivariate logistic regression models were used in association analyses. Survival analyses were performed using log-rank and Cox-proportional hazards models.

To assess the value of mtDNA level in risk prediction, we compared bivariate and multivariate models with and without mtDNA level using the net reclassification improvement approach [30] (using risk categories in increments of 10% from 0% to 100%). In contrast to c-statistics, the net reclassification index (NRI) may have unique value in assessing the ability of a biomarker to improve risk prediction when groups can be split up into risk strata. All analyses were performed using Statistical Analysis Software version 9.2 (SAS Institute). Analyses comparing c-statistics and net reclassification were performed using rocplus [31]. *p*<0.05 was considered statistically significant.

## Results

### Baseline Characteristics

Baseline characteristics of ICU patients included in the BWH RoCI (*n* = 200) and the ME ARDS (*n* = 243) cohorts are presented in Table 1. While the BWH RoCI cohort was entirely composed of MICU patients, the ME ARDS cohort included patients from different ICU types. Patients in both studies were more likely to be white, to be female, and to carry a diagnosis of sepsis; however, these covariates were even more prevalent among patients in the ME ARDS cohort. A diagnosis of ARDS was more common in ME ARDS patients than in BWH RoCI patients. Age, APACHE II scores, and mtDNA levels were similar in both studies. Of note, only one patient in either study (in ME ARDS) received activated protein C during their stay in the ICU.

**Table 1:** Baseline characteristics of the ICU patients.

Category	Variable	Number (Percent) or Median (Interquartile Range)	
		BWH RoCI (*n* = 200)	ME ARDS (*n* = 243)
**Demographic parameters**	**Age (years)**	60 (46–69)	59 (45–71)
	**Gender (female)**	117 (59%)	158 (65%)
	**Race/ethnicity**		
	White	155 (78%)	219 (90%)
	African-American	26 (13%)	5 (2%)
	Hispanic	13 (7%)	14 (6%)
	Asian or Pacific Islander	6 (3%)	5 (2%)
**Clinical parameters**	**ICU type**		
	Medical	200 (100%)	126 (52%)
	Cardiac		26 (11%)
	Surgical		82 (34%)
	Trauma		7 (3%)
	Neurological		2 (1%)
	**Cancer**		
	Any	89 (45%)	—
	Metastatic solid tumor	—	4 (2%)^a^
	**APACHE II score**	24 (18–30)	22 (18–26)
	**Acute kidney injury** b	57 (29%)^a^	—
	**Need for mechanical ventilation**	90 (45%)^a^	—
	**Heart failure** c	17 (9%)^a^	—
	**Liver failure** c	20 (10%)^a^	10 (4%)^a^
	**Vasopressor use**	71 (36%)^a^	—
	**Sepsis**	124 (62%)	187 (77%)
	**ARDS**	30 (15%)	104 (43%)^a^
**Mortality data**	**28-d mortality**	60 (30%)	40 (16%)
	**In-hospital morality**	51 (26%)	—
	**Overall mortality**	93 (47%)	—
**Peripheral blood biomarkers of ICU mortality**	**mtDNA (copies/µl)** d	3,379 (1,123–12,227)	3,544 (1,130–10,557)
	**Procalcitonin (µg/l)**	3.6 (0.9–12.1)^a^	—
	**Lactate (mg/dl)**	1.9 (1.2–4.0)^a^	—

^a^ Data missing in BWH RoCI cohort for liver failure in one patient (0.5%), for acute kidney injury, need for mechanical ventilation, and heart failure in two patients (1%), for vasopressor use in four patients (2%), for procalcitonin measurement in 54 patients (27%), and for lactate measurement in 94 patients (47%). Data missing in the ME ARDS cohort for the presence of a solid tumor with metastasis and for liver failure in two patients (1%), and in all patients for particular variables, where indicated.

^b^ Acute kidney injury defined by a ≥0.5 mg/dl increase in serum creatinine [17].

^c^ Based on admitting physician report.

^d^ mtDNA level represents NADH dehydrogenase 1 DNA level expressed as copies per microliter.

The additional analysis of patient characteristics is shown in Text S4 in Supporting Information S1.

Of the 200 patients in the BWH RoCI study, 60 (30%) died within 28 d of MICU admission, and 51 (25%) died in the hospital. The median follow-up time for the group that died in the hospital was 5 d (interquartile range 1–54 d). In the ME ARDS study, 40 patients (16%) died within 28 d of ICU admission. In the ME ARDS study, the rate of death among MICU patients (i.e., medical or cardiac ICU patients, *n* = 31 deaths, 20%) differed from that among non-MICU patients (i.e., surgical, trauma, or neurological ICU patients, *n* = 9 deaths, 10%, *p* = 0.03 for the difference). A majority of plasma samples were drawn within 4 d of ICU admission in both the BWH RoCI (*n* = 186, 93%) and the ME ARDS (*n* = 194, 90%, data missing on 34 patients) studies.

### mtDNA Level and Mortality

#### BWH RoCI

**Figure 2:**
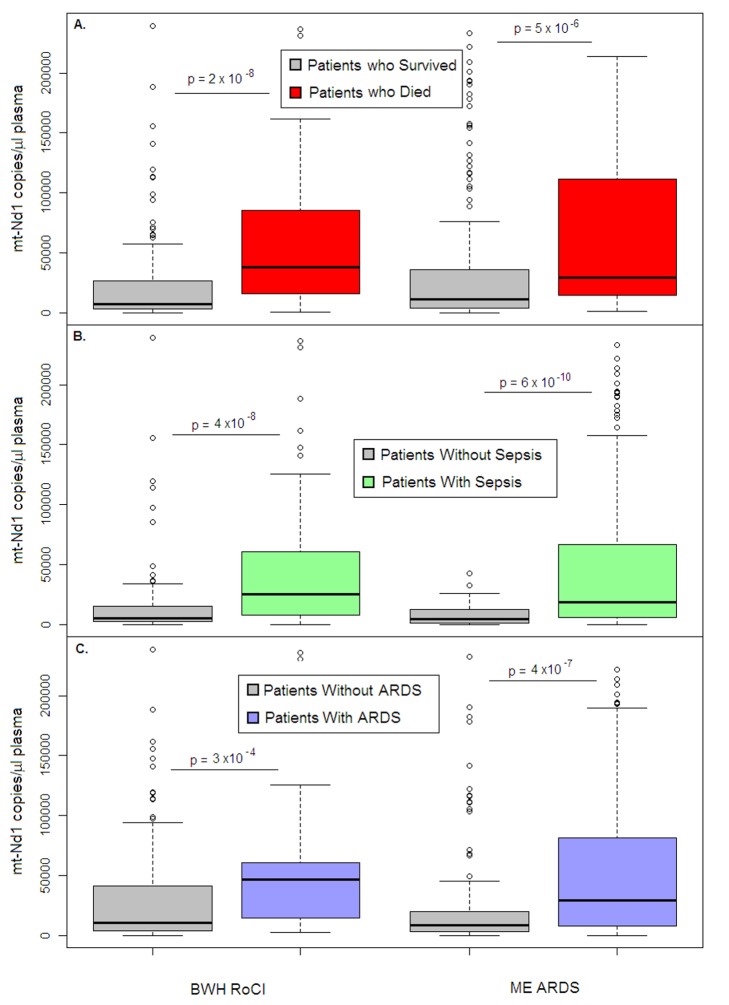
Cell-free mtDNA level in the plasma of ICU patients. Boxplots comparing measures of cell-free mtDNA level (mtDNA [NADH dehydrogenase 1] expressed as copy number/µl of plasma) in the plasma of (A) patients who died within 28 d after ICU admission (red) versus those who survived (gray), (B) patients with (green) versus without (gray) sepsis, and (C) patients with (blue) versus without (gray) ARDS in the BWH RoCI (left) and ME ARDS (right) cohorts. mtDNA copy number in the plasma is presented as median value (black line), interquartile range (box), and 5th and 95th percentiles (whiskers). *p*-Values are noted in the figure for each cohort.

In the BWH RoCI cohort, mtDNA level was higher in ICU patients who died within 28 d of MICU admission than in those who did not (median 9,504 copies/µl versus 1,927 copies/µl, *p* = 2×10^−8^; Figure 2A), as well as in patients with diagnoses commonly associated with mortality in the ICU such as sepsis (Figure 2B) and ARDS (Figure 2C). For each log_10_-unit increase in mtDNA level, patients had an increased odds of dying within 28-d of MICU admission (odds ratio [OR] 1.9, 95% CI 1.5–2.4, *p* = 2×10^−7^). Patients with an mtDNA level ≥3,200 copies/µl had an increased odds of dying within 28 d of MICU admission (Table 2) and an increased odds of dying in the hospital (OR 8.4, 95% CI 3.7–19.2, *p* = 4×10^−7^). Of those who died within 28 d of MICU admission, 80% (*n* = 48 of 60) had an mtDNA level ≥3,200 copies/µl compared to 38% (*n* = 53 or 140) of those who did not die. In the BWH RoCI cohort, the odds of dying within 28 d of MICU admission was higher among those with an mtDNA level ≥3,200 copies/µl when the level was obtained <48 h after ICU admission (*n* = 52, OR 23.0, 95% CI 4.4–120.0, *p* = 2×10^−4^) compared to those with an mtDNA level ≥3,200 copies/µl that was obtained ≥48 h after ICU admission (*n* = 148, OR 4.4, 95% CI 1.9–9.8, *p* = 4×10^−4^); however, this difference was of borderline statistical significance (mtDNA by ICU status interaction *p* = 0.08). The additional analysis of nuclear DNA level and mtDNA level in different patient groups in the BWH RoCI study is shown in Texts S5 and S6 in Supporting Information S1.

**Table 2:** Bivariate and multivariate analyses of association between elevated mtDNA levels and 28-d MICU mortality.

Model	28-d MICU Mortality			
	BWH RoCI(*n* = 200)	ME ARDS		
		All Patients(*n* = 243)	MICU Patients^a^(*n* = 152)	Non-MICU Patients^b^(*n* = 91)
Unadjusted	6.6 (3.2–13.4)3×10^−7^	3.8 (1.7–8.3)0.001	5.3 (2.0–14.0)6×10^−4^	1.7 (0.4–7.4)0.46
Adjusted^c^	7.5 (3.6–15.8)1×10^−7^	4.2 (1.9–9.6)6×10^−4^	8.4 (2.9–24.2)9×10^−5^	1.5 (0.3–7.0)0.58
Adjusted^c^+APACHE II score	5.6 (2.6–12.1)1×10^−5^	3.4 (1.4–8.4)0.008	6.2 (1.9–20.4)0.002	1.1 (0.2–5.6)0.90
Adjusted^c^+sepsis	9.0 (3.9–20.7)2×10^−7^	3.1 (1.4–7.3)0.008	6.0 (2.0–17.8)0.001	1.1 (0.2–5.5)0.92
Adjusted^c^+ARDS	6.9 (3.3–14.8)5×10^−7^	3.6 (1.2–10.8)0.02	5.3 (1.6–17.5)0.007	1.3 (0.3–6.1)0.73

Data are given as OR (95% CI) followed by *p*-value. Elevated mtDNA level represents NADH dehydrogenase 1 DNA copy number of ≥3,200 copies/µl plasma.

^a^ Includes MICU and cardiac ICU patients.

^b^ Includes surgical ICU, trauma ICU, and neurological ICU patients.

^c^ Multivariate models in both cohorts were adjusted for age, gender, and race/ethnicity, with additional adjustments where indicated.

There was no evidence in the BWH RoCI cohort that the association between mtDNA level and MICU mortality was attenuated in models adjusted for clinical covariates, including APACHE II score, sepsis, or ARDS (Table 2). There was no statistical evidence that any of these variables significantly modified the effect of mtDNA level on mortality.

While an elevated mtDNA level was associated with acute kidney injury (OR 3.2, 95% CI 1.7–6.3, *p* = 4×10^−4^), the need for mechanical ventilation (OR 2.7, 95% CI 1.5–4.8, *p* = 7×10^−4^), the use of vasopressors (OR 3.6, 95% CI 1.9–6.6, *p* = 6×10^−5^), and an underlying diagnosis of cancer (OR 1.8, 95% CI 1.0–3.1, *p* = 0.05), there was no significant association of elevated mtDNA with physician report of either liver failure (*p* = 0.65) or heart failure (*p* = 0.45). The association between an elevated mtDNA level and 28-d mortality remained significant in the BWH RoCI cohort even after adjustment for additional variables including use of vasopressors, need for mechanical ventilation, cancer, acute kidney injury, or admitting physician impression of either heart or liver failure (OR 9.5, 95% CI 3.5–25.6, *p* = 3×10^−5^; see Table S3 in Supporting Information S1).

Binary classification analysis for elevated mtDNA levels and 28-d MICU mortality are shown in Table 3.

**Table 3:** Elevated mtDNA levels and 28-d MICU mortality by group.

Statistical Test	28-d MICU Mortality			
	BWH RoCI(*n* = 200)	ME ARDS		
		All Patients(*n* = 243)	MICU Patients^a^(*n* = 152)	Non-MICU Patients^b^(*n* = 91)
Sensitivity	80%	75%	81%	67%
Specificity	62%	52%	56%	46%
Positive predictive value	48%	24%	32%	12%
Negative predictive value	88%	92%	92%	93%

Elevated mtDNA level represents NADH dehydrogenase 1 DNA copy number of ≥3,200 copies/µl plasma.

^a^ Includes MICU and cardiac ICU patients.

^b^ Includes surgical ICU, trauma ICU, and neurological ICU patients.

Of the 200 patients from the BWH RoCI cohort, we were able to obtain simultaneous measurements of procalcitonin in 146 (73%) and lactate in 106 (53%) patients. To determine whether the association between mtDNA and 28-d mortality could be explained by measures of procalcitonin or lactate, we tested each of these biomarkers for association with mortality and then assessed whether adjusting for either procalcitonin or lactate attenuates the association between mtDNA and mortality. All three biomarkers were significantly associated with 28-d mortality (Table 4). mtDNA level remained significantly associated with 28-d mortality even after adjusting for either procalcitonin or lactate. In contrast, the associations between procalcitonin and lactate and mortality were attenuated in models additionally adjusting for mtDNA level (Table 4).

**Table 4:** Peripheral blood biomarkers and 28-d ICU mortality.

Association		Prediction			
BWH RoCI (*n* = 200)		BWH RoCI (*n* = 200)		ME ARDS	
				All Patients(*n* = 243)	MICU Patients^a^(*n* = 152)
Model	OR (95% CI)*p*-Value	Model	NRI (SE)*p*-Value^b^	NRI (SE)*p*-Value^b^	NRI (SE)*p*-Value^b^
Elevated mtDNA level^c^	6.6 (3.2–13.4)3×10^−7^	Elevated mtDNA level^c^+clinical model^d^	79% (14%)<0.0001	28% (13%)0.03	55% (20%)0.007
Procalcitonin (µg/l)	1.02 (1.01–1.03)0.004	Procalcitonin (µg/l)+clinical model^d^	11% (9%)0.25	—	—
Lactate (mg/dl)	1.4 (1.1–1.7)0.002	Lactate (mg/dl)+clinical model^d^	36% (15%)0.02	—	—
Elevated mtDNA level^c^+procalcitonin (µg/l)^e^	6.6 (2.3–18.2)5×10^−4^	Elevated mtDNA level^c^+clinical model^d^+procalcitonin (µg/l)	52% (15%)6×10^−4^	—	—
Elevated mtDNA level^c^+lactate (mg/dl)^f^	7.0 (2.1–22.8)0.001	Elevated mtDNA level^c^+clinical model^d^+lactate (mg/dl)	36% (17%)0.03	—	—

^a^ Includes MICU and cardiac ICU patients.

^b^ *p*-Value using risk categories in increments of 10% from 0%–100%. In all cases the NRI value refers to the addition of the noted biomarker to the clinical model (including age, gender, race/ethnicity, APACHE II score, and sepsis) and additional biomarkers, where indicated.

c Elevated mtDNA level represents NADH dehydrogenase 1 DNA copy number of ≥3,200 copies/µl plasma compared to those with <3,200 copies/µl.

d Clinical model includes age, gender, race/ethnicity, APACHE II score [20], and sepsis.

e The effect of procalcitonin on the mortality is no longer significant after adjusting for an elevated mtDNA level (OR 1.01, 95% CI 0.99–1.02, *p* = 0.42).

f The effect of lactate on the mortality is attenuated after adjusting for an elevated mtDNA level (OR 1.2, 95% CI 0.99–1.51, *p* = 0.05).

Of the 200 BWH RoCI patients, 96 (48%) remained in the ICU for 7 d and had a repeat measurement of mtDNA. Mortality level was lowest among patients with mtDNA levels <3,200/µl copies at both their initial blood draw and day 7 (0% mortality), highest in those with levels ≥3,200/µl copies at both visits (39% mortality), and intermediate for those with an elevated mtDNA level at only one of the two time points (∼12% mortality; see Table 5).

**Table 5:** Repeated measures of mtDNA and 28-d mortality in the BWH RoCI cohort.

Categories Based on Repeated mtDNA Measurement	Number (Percent)^a^ Alive at 28-d (*n* = 74)	Number (Percent)^a^ Who Died after 28-d (*n* = 22)
mtDNA level <3,200 copies/µl on initial blood draw and day 7	10 (100%)	0 (0%)
mtDNA level ≥3,200 copies/µl on initial blood draw and <3,200 copies/µl on day 7	7 (87.5%)	1 (12.5%)
mtDNA level <3,200 copies/µl on initial blood draw and ≥3,200 copies/µl on day 7	30 (88%)	4 (12%)
mtDNA level ≥3,200 copies/µl on initial blood draw and day 7	27 (61%)	17 (39%)^c^

mtDNA level represents NADH dehydrogenase 1 DNA copy number.

a Data missing for 104 (52%) of the BWH RoCI patients at day 7 of ICU admission. The mortality rate was higher among those missing data at day 7 of ICU admission (37%, *n* = 38) than among those who had data at day 7 of ICU admission (23%, *n* = 22, *p* for the comparison = 0.03).

c There was a significant association between repeated measures of mtDNA and mortality among these BWH RoCI patients (Fisher's exact test, *p* = 0.007).

**Figure 3:**
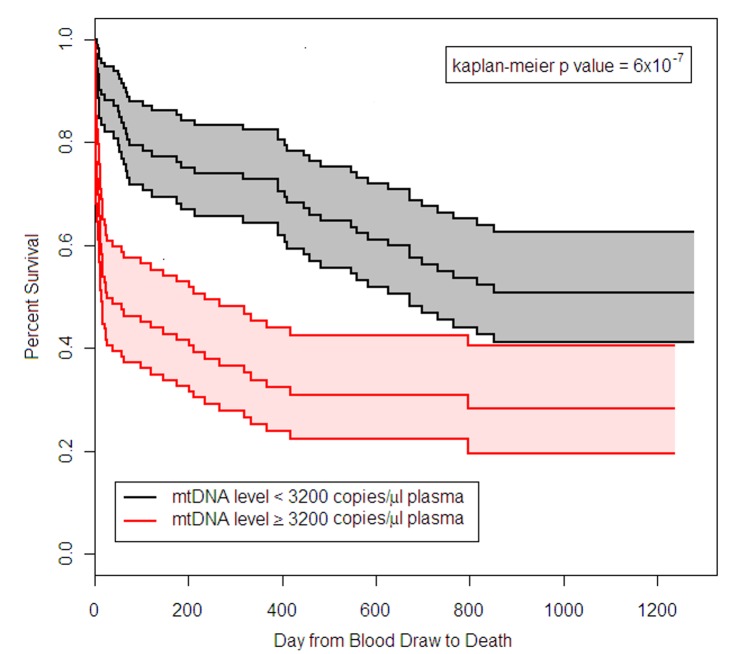
Survival of BWH RoCI MICU patients stratified by mtDNA level. Kaplan-Meier estimates of survival for patients who had plasma mtDNA levels ≥3,200 copies/µl (*n* = 101) and <3,200 copies/µl (*n* = 99) in the BWH MICU. The middle black line indicates the Kaplan-Meier survival curve for patients who had mtDNA <3,200 copies/µl, with 95% confidence intervals (outer black lines and gray shading). The middle red line indicates the Kaplan-Meier survival curve for patients who had mtDNA ≥3,200 copies/µl, with 95% confidence intervals (outer red lines and pink shading). Survival in patients with mtDNA ≥3,200 copies/µl was significantly lower than in patients with mtDNA <3,200 copies/µl (*p*-value noted in the figure).

Follow-up data were available on the patients in the BWH RoCI study for periods ranging from 1 d to 3.5 y after admission to the ICU. 54% (*n* = 108) of the cohort died during follow-up. Patients who had an mtDNA level ≥3,200 copies/µl had an increased risk of death (hazard ratio 2.4, 95% CI 1.6–3.6, *p*<1×10^−4^; see Figure 3). There was no evidence that the effect of mtDNA level on the risk of death was attenuated in adjusted models (Text S7 in Supporting Information S1). mtDNA level was associated with an increased risk of death even in analyses limited to patients with sepsis (after adjusting for age, gender, race/ethnicity, and APACHE II score, an mtDNA level ≥3,200 copies/µl was associated with an increase in the risk of death, hazard ratio 3.0, 95% CI 1.5–6.3, *p* = 0.003) or ARDS (after adjusting for age, gender, race/ethnicity, and APACHE II score, an mtDNA level ≥3,200 copies/µl was associated with a 2.3-fold increase in the risk of death, hazard ratio 2.3, 95% CI 1.5–3.5, *p* = 2×10^−4^).

#### ME ARDS

Comparable to our findings in the BWH RoCI cohort, mtDNA levels were higher in ME ARDS patients who died within 28 d of ICU admission (median 7,457 copies/µl) than in patients who did not (median 2,846 copies/µl, *p* = 5×10^−6^; Figure 2A), and were higher in patients with sepsis (Figure 2B) or ARDS (Figure 2C) than in patients without these conditions. Also similarly to the BWH RoCI cohort, for each log_10_-unit increase in mtDNA level, patients had an increased odds of dying within 28-d of ICU admission (OR 1.6, 95% CI 1.2–2.0, *p* = 9×10^−4^), those with an mtDNA level ≥3,200 copies/µl had an increased odds of dying within 28 d of ICU admission (Table 2), and there was no evidence that the association between mtDNA level and ICU mortality was attenuated in models adjusted for covariates (Table 2).

In contrast to the BWH RoCI study, the ME ARDS study included ICU patients from non-medical ICUs (i.e., surgical, trauma, and neurological ICUs). In stratified analyses, the association between an elevated mtDNA level and 28-d mortality was limited to MICU patients. Importantly, there was no evidence for an association between mtDNA level and 28-d mortality in non-MICU patients (mtDNA by ICU status interaction 0.03; see Table 2).

### mtDNA Level and 28-d Mortality Reclassification and Prediction

#### BWH RoCI

**Figure 4:**
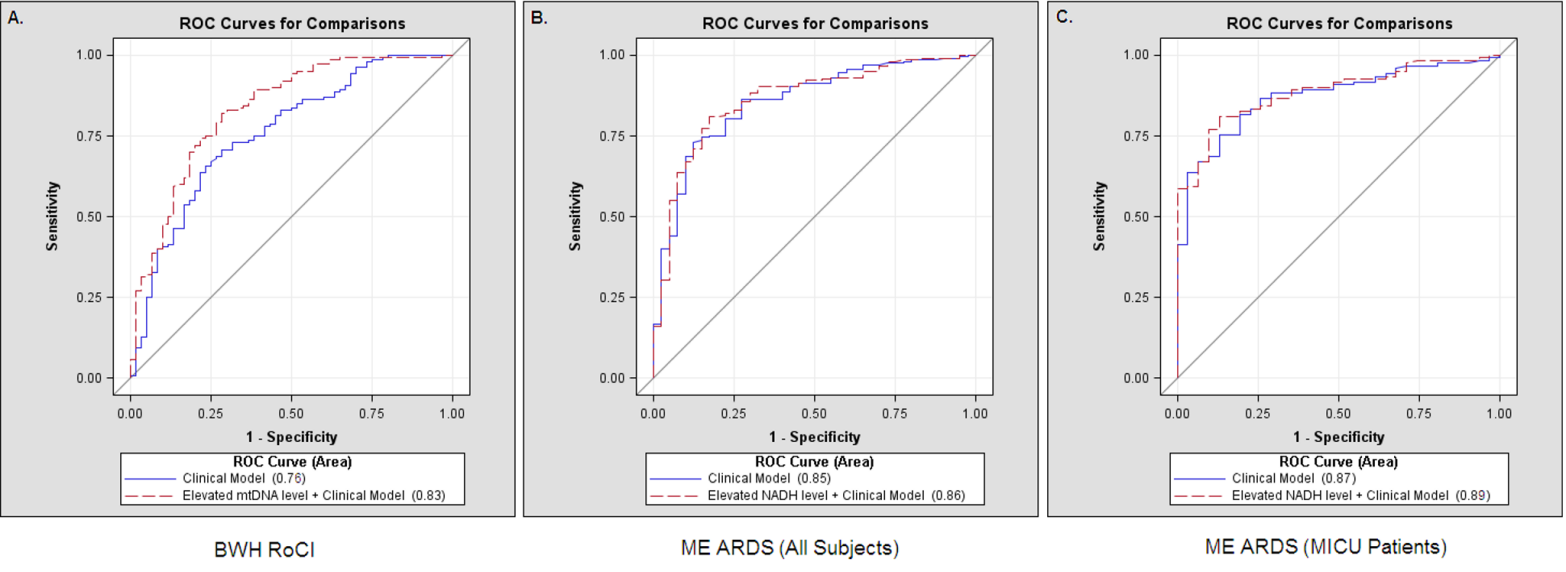
Receiver operating characteristic curves, mtDNA, and death in ICU patients. Comparisons of receiver operating characteristic (ROC) curves for a clinical model (including age, gender, race/ethnicity, APACHE II score, and sepsis) (solid lines) and a clinical model with an mtDNA level ≥3,200 copies/µl (dashed lines) to predict 28-d mortality in ICUs. (A) The area under the curve was 0.76 for a clinical model and was 0.83 for a clinical model with an mtDNA level ≥3,200 copies/µl in the BWH RoCI cohort. (B) The area under the curve was 0.85 for a clinical model and was 0.86 for a clinical model with an mtDNA level ≥3,200 copies/µl in the ME ARDS cohort when all patients were included. (C) The area under the curve was 0.87 for a clinical model and was 0.89 for a clinical model with an mtDNA level ≥3,200 copies/µl in the subpopulation of ME ARDS MICU patients.

To further assess the importance of mtDNA as an ICU biomarker, we tested the ability of mtDNA level to improve reclassification of patients in the BWH RoCI cohort. The inclusion of mtDNA level ≥3,200 copies/µl resulted in a 79% improvement in net reclassification when added to a clinical model (including age, gender, race/ethnicity, APACHE II score, and sepsis; see Table 4). The improvement in net reclassification remained significant, albeit attenuated, when mtDNA level was added to clinical models including known ICU biomarkers (e.g., procalcitonin or lactate; see Table 4). While measurement of lactate levels improved the reclassification of patients when added to a clinical model, measurement of procalcitonin did not (Table 4). In contrast, measurement of lactate did not improve reclassification when added to a clinical model that included mtDNA level (NRI 4%, standard error [SE] 14%, *p* = 0.80). Similarly, in the BWH RoCI cohort there was evidence that an mtDNA level of ≥3,200 copies/µl improved 28-d mortality prediction when added to a model with clinical variables using c-statistics (the c-statistic was 0.76, 95% CI 0.68–0.83, for a model including age, gender, race/ethnicity, APACHE II score, and sepsis, which improved to 0.83, 95% CI 0.77–0.90, with the inclusion of mtDNA level, *p*<0.001 for the comparison) (Figure 4A).

#### ME ARDS

Similar to our findings in the BWH RoCI cohort, the addition of an elevated mtDNA level improved net reclassification of ICU patients when added to a clinical model in the ME ARDS cohort (Table 4). The improvement in net reclassification in the ME ARDS cohort was due to the improvement in reclassifying MICU patients (Table 4), as no significant improvement was noted in non-MICU patients (NRI 4%, SE 16%, *p* = 0.82). Similarly, there was evidence that an mtDNA level of ≥3,200 copies/µl improved 28-d mortality prediction when added to a model with clinical variables using c-statistics (the c-statistic was 0.85, 95% CI 0.79–0.92, for a model including age, gender, and APACHE II score, which improved to 0.86, 95% CI 0.80–0.92, with the inclusion of mtDNA level, *p* = 0.02 for the comparison) in the ME ARDS cohort (Figure 4B), although improvement in prediction was limited to MICU patients (the c-statistic was 0.87, 95% CI 0.81–0.93, for a model including age, gender, and APACHE II score, which improved to 0.89, 95% CI 0.83–0.94, with the inclusion of mtDNA level, *p* = 0.008 for the comparison) (Figure 4C), as no significant improvement was noted in non-MICU patients (the c-statistic was 0.82 regardless of the inclusion of mtDNA level, *p* = 0.97 for the comparison).

Additional analyses combining both cohorts are presented in Text S8 and Table S4 in Supporting Information S1.

## Discussion

Elevated levels of circulating cell-free mtDNA are associated with ICU mortality, particularly among MICU patients, from multiple cohorts. We demonstrate that mtDNA can improve risk prediction beyond commonly measured biomarkers, such as lactate, and combined measures of baseline characteristics and underlying physiology (e.g., APACHE II score) among MICU patients. In the BWH RoCI cohort, based primarily on the strength of the association between elevated mtDNA level and 28-day mortality, elevated mtDNA levels predicted an increased risk of death that persisted over up to 3.5 y of follow-up.

Based on the data we present in this study, it is not possible to determine whether the observed increased circulating cell-free mtDNA levels were increased as the result of the severity of illness alone, or whether mtDNA actively contributes to disease pathogenesis in MICU patients [32]–[34]. Recent observations [12] show that mtDNA contributes to activation of inflammasome, a large multimolecular complex that controls the proteolytic enzyme caspase-1 [33],[34]. Inflammasome activation is exclusively responsible for caspase-1-mediated IL-1β and IL-18 maturation and secretion in immune cells [33],[34], and circulating IL-1β and IL-18 levels are known to be increased in critically ill patients with both sepsis and ARDS [1],[14],[35]. These findings, in conjunction with the findings presented in here, suggest that circulating mtDNA could serve as a marker of death and, independently, may represent an important pathogenic determinant that contributes to an exaggerated systemic inflammatory response [36].

Our data are consistent with prior studies demonstrating that circulating cell-free mtDNA levels are elevated in critically ill subpopulations including adults with community-acquired bacterial meningitis [8], trauma [7], or sepsis in the emergency room [37], and further implicates the mitochondria, both in terms of genotyping (mtDNA haplogroups) [38] and now cell-free mtDNA plasma measurement, as an important factor predicting survival. In addition, our study demonstrates, to our knowledge for the first time, that elevated mtDNA levels may be particularly important in improving risk prediction in the MICU.

In addition to the evidence that elevated mtDNA levels are associated with sepsis, ARDS, and mortality, an important and unique aspect of our study is that we provide replicated evidence that mtDNA level improves risk prediction in MICU patients. Metrics of association and prediction should not be confused, as only the latter suggest that a finding may have clinical applicability as a test for an individual patient [39]. Although we present statistical evidence that mtDNA level improves our ability to predict death among MICU patients using both c-statistics and reclassification methods, it is important to note that some studies suggest that reclassification methods are more sensitive than c-statistics when comparing the magnitude of predictive improvement [30],[40].

Our study has a number of important limitations. First, although we demonstrate that an elevated mtDNA measurement is associated with ICU mortality among MICU patients from multiple cohorts, there was no evidence for a similar association among non-medical (predominantly surgical) ICU patients. Although we cannot definitively determine the reasons behind these differences within the context of this study, we can't rule out that subtle differences in the timing of sample collection could have influenced the associations within these two groups. In addition, these findings suggest that mtDNA could be released from cells or tissue as the result of surgery, and in this situation an elevated mtDNA level may be unlikely to be associated with an increased risk of mortality. Of note, although some studies demonstrate that patients who meet criteria for systemic inflammatory response syndrome (SIRS) have worse outcomes [41], this does not appear to hold among surgical ICU patients, where the presence of systemic inflammatory response syndrome is very common [42]. Second, some of the comparisons between biomarkers in the BWH RoCI study are limited because of missing data (e.g., lactate measurements). Third, although our assessments of repeated measures of mtDNA suggest that the trajectory of this measurement may be of clinical importance, we urge caution in interpreting these results, as our findings also demonstrate that the probability of being alive to have had mtDNA remeasured was also dependent on the initial mtDNA level. These findings support the idea that future studies of mtDNA in MICU patients could benefit from repeated measurements, including those prior to ICU presentation. Fourth, although fungemia was rare in both the BWH RoCI and ME ARDS cohorts (Text S4 in Supporting Information S1), it is worth noting that fungi have mtDNA that may cross-react with human mtDNA primers [10],[43]. Finally, it is important to note that our analyses reflect that the mtDNA level adds to, rather than replaces, information gleaned from commonly measured physiologic parameters and biomarkers.

In conclusion, we show that mtDNA level in plasma is associated with mortality in MICU patients. Elevated mtDNA level adds to mortality risk prediction in the ICU, and represents an advance over currently used ICU biomarkers and the APACHE score. Given the relative ease and rapidity (the entire process currently takes <6 h) of mtDNA measurement, circulating cell-free mtDNA could be a valuable addition to assessment of MICU patients, and points the way to the possibility of new diagnostic and/or therapeutic approaches for patients with critical illness.

## Supporting Information

Supporting Information S1 **mtDNA levels and mortality in patients in the ICU.**
(DOCX)

Figure S1 **Plot of the threshold cycle (Ct) against the input DNA concentration of the samples after the qPCR using bacterial DNA.** DNA isolated from *Escherichia coli* (Gram negative bacteria), *Enterococcus faecalis* (Gram positive bacteria), and *Clostridium difficile* (anaerobic bacteria) were subject to qPCR analysis using primers for human mtDNA primers or bacterial 16S ribosomal RNA (bacterial 16S). While bacterial 16S primers amplified DNA from each of our bacterial samples, human mtDNA primers were not able to amplify DNA from any bacterial sample.
(TIF)

Checklist S1 **STARD checklist.**
(DOC)

## Abbreviations

APACHE: Acute Physiology and Chronic Health Evaluation
ARDS: acute respiratory distress syndrome
BWH RoCI: Brigham and Women's Hospital Registry of Critical Illness
ICU: intensive care unit
ME ARDS: Molecular Epidemiology of Acute Respiratory Distress Syndrome
MICU: medical intensive care unit
mtDNA: mitochondrial DNA
NRI: net reclassification index
OR: odds ratio
qPCR: quantitative real-time polymerase chain reaction
SE: standard error
