# Supplementary material for: Circulating Mitochondrial DNA in Patients in the ICU as a Marker of Mortality: Derivation and Validation
Source: PLoS Med. 2013 Dec 31;10(12):e1001577. doi: 10.1371/journal.pmed.1001577 (PMC3876981; doi:10.1371/journal.pmed.1001577)
Supplement: Supporting Information S1 — mtDNA levels and mortality in patients in the ICU. (DOCX) [file pmed.1001577.s001.docx]

Supporting Information for

**Circulating Mitochondrial DNA in Patients in the ICU as a Marker of Mortality: Derivation and Validation**

Kiichi Nakahira, Sun-Young Kyung, Angela J. Rogers, Lee Gazourian, Sojung Youn, Anthony F. Massaro, Carolina Quintana, Juan C. Osorio, Zhaoxi Wang, Yang Zhao, Laurie A. Lawler, Jason D. Christie, Nuala J. Meyer, Finnian R. Mc Causland, Sushrut S. Waikar, Aaron B. Waxman, Raymond T. Chung, Raphael Bueno, Ivan O. Rosas, MICU Registry, Laura E. Fredenburgh, Rebecca M. Baron, David C. Christiani, Gary M. Hunninghake, Augustine M. K. Choi.

To whom correspondence should be addressed to: [amc2056@med.cornell.edu](mailto:amc2056@med.cornell.edu)

• Supporting Information TEXT

Text S1: Subject recruitment.

Text S2: Assessment for specificity of mtDNA primers.

Text S3: BLAST search for mtDNA primers.

Text S4: Additional patient characteristics.

Text S5: MtDNA level and Selected Patient Characteristics in the BWH RoCI.

Text S6: Nuclear DNA level and in hospital mortality.

Text S7: MtDNA level and overall mortality in the BWH RoCI.

Text S8: MtDNA level and 28-day mortality in combined analyses.

• Figure S1: The amplification plots (ΔRn vs. Cycle (log) view) after the qPCR using bacterial DNA were shown.

• Table S1: BLAST search for bacterial genome.

• Table S2: BLAST search for human genome.

• Table S3: Multivariate Logistic Regression Analysis of Association of the Odds for 28- day Mortality in the BWH RoCI.

• Table S4: Combined analyses of an Elevated MtDNA Levels and 28-day MICU Mortality.

• References for Supporting Information

**Text S1:**

Subject Recruitment

BWH RoCI: The Brigham and Women’s Hospital (BWH) Research Registry and Human Sample Repository for the Study of the Biology of Critical Illness (BWH RoCI), in an ongoing registry that collects demographic, clinical information, and blood specimens from patients admitted in the medical intensive care unit (MICU). BWH RoCI is approved by the Partners Human Research Committee and operates under protocol 2008-P-000495. All adults (age 18 and older) admitted to the MICU to the BWH are considered for enrollment. Exclusion criteria included, participants with 1) mental handicaps, 2) who are unable to provide consent or for whom an appropriate legal representative could not be found to provide consent, 3) who had previously indicated that they did not wish to be enrolled in this study (e.g. during a prior admission to the MICU), 4) who were admitted for comfort measures and, 5) for whom there was concern about non-medically indicated blood draws (e.g. Jehovah’s witnesses).

ME ARDS: Consecutive admissions to the ICUs were screened. Patients with predisposing conditions for ARDS, including bacteremia, sepsis, pneumonia, trauma, aspiration, or multiple transfusions as defined previously, were eligible for inclusion. Exclusion criteria were aged <18 years, diffuse alveolar hemorrhage, chronic lung diseases other than COPD or asthma, directive to withhold intubation, immunosuppression (other than immunosuppression secondary to corticosteroid therapy), and treatment with granulocyte colony-stimulating factor. Enrolled patients were followed daily for ARDS development on the basis of the American-European Consensus Committee criteria for ARDS.

Racial and Ethnic Category:

The racial and ethnic category is taken from the medical record, and this information is self-identified by the patient or family.

Diagnosis:

SIRS and sepsis was identified according to the 2001 SCCM/ESICM/ACCP/ATS/SIS International Sepsis Definitions Conference guidelines [1]. ARDS was defined according to American-European Consensus Conference on ARDS guidelines [2].

**Control:** Patients requiring MICU hospitalization without known or suspected infectious origin.

**SIRS:** 2 or more of the following criteria:
- Temperature < 36 °C or > 38 °C
- HR > 90
- RR > 20 or PaCO_2_ < 32
- WBC > 12000  < 4000, or > 10% immature (band) forms

**Sepsis:** Documented infection (culture positive or strong clinical suspicion) together with 2 or more SIRS criteria above.

**ARDS:** ALI requiring all four of the following features in patients who have a risk factor for ARDS and no history of chronic lung disease: Acute onset, Bilateral infiltrates (radiographically similar to pulmonary edema), No evidence of elevated left atrial pressure (the pulmonary capillary wedge pressure is ≤18 mmHg if measured), A ratio of arterial oxygen tension to fraction of inspired oxygen (PaO2/FiO2) <200 mmHg.

**Text S2:**

Assessment for specificity of mtDNA primers

To assess whether our mtDNA primers cross-react with bacterial genomes, we performed qPCR using our mtDNA primers and isolated bacterial DNA from *Escherichia coli* (Gram negative bacteria), *Enterococcus faecalis* (Gram positive bacteria) and *Clostridium difficile* (Anaerobic bacteria). As shown in **Figure S1**, although bacterial 16s ribosomal RNA primers amplified DNA from each of our bacterial samples, our mtDNA primers were not able to amplify DNA from any bacterial samples.

**Text S3:**

BLAST search for mtDNA primers

To assess the identical or similar sequence between the primer sequences and bacterial DNA sequences, we perform microbial BLAST search and calculated the expect value (E value) by using National Center for Biotechnology Information (NCBI) database. E value is an important a parameter that describes the number of hits one can "expect" to see by chance when searching a database of a particular size. It decreases exponentially as the Score of the match increases. Essentially, the E value describes the random background noise. The lower the E-value, or the closer it is to zero, the more "significant" the match is. (http://blast.ncbi.nlm.nih.gov/Blast.cgi?CMD=Web&PAGE_TYPE=BlastDocs&DOC_TYPE=FAQ#expect) While the lowest E value was 1.3 in genome of *Oceanithermus profundus* (for forward mtDNA primer) and *Rhodanobacter sp.* (for reverse mtDNA primer), E values calculated in other bacteria were over 5.0 in the BLSAT search for bacterial genome (**Table S1**). We listed below top ten bacterial with the lowest E values in the blast search using our mtDNA primer sequences (Human mtDNA) and previously reported primer sequences for detecting human mitochondrial DNA (Human cytochrome B forward, 5’- ATGACCCCAATACGCAAAAT-3’; reverse, 5’-CGAAGTTTCATCATGC GGAG-3’) [3,4] (**Table S1**). We also performed BLAST search for human genome by using sequences of our mtDNA primers and the human cytochrome B primers (**Table S2**). Importantly, E value was 0.021929 for human mitochondrial genome in the BLAST search using our mtDNA primers (both forward and reverse) and was the only human genome with the E value below 1.0, while there are several human nuclear DNA sequences with low E values for the human cytochrome B primers (**Table S2**).

**Text S4:**

Additional patient characteristics

The study inclusion was made on the ICUs admission in both cohorts. In the BWH RoCI cohort, we found that 36 (18%) of patients came from outside of BWH, 38 (19%) were transferred in BWH, and 126 (63%) came from the Emergency Department of BWH. In ME ARDS, we found that 45 (19%) of the patients were from the operation rooms or the recover rooms in MGH or BIDMC, 99 (40%) were from the emergency rooms, 51 (21%) were transferred in the hospitals, and 48 (20%) came from outside of the hospitals.

We also found that five patients out of 200 patients had candidemia in RoCI, and two patients out of 243 patients had candidemia in ME ARDS.

**Text S5:**

MtDNA level and Selected Patient Characteristics in the BWH RoCI

Of the 29 controls in the BWH RoCI, 4 (14%) had an initial mtDNA level >3200 copies/μl. These 4 patients were admitted with varying diagnoses including multiple myeloma with amyloidosis, squamous cell carcinoma of the tongue with an aspiration pneumonitis (this patient died within 28-days of ICU admission), a patient with chronic renal insufficiency and superior vena cava syndrome secondary to a central line associated thrombosis, and a patient with a COPD exacerbation.

Of the 60 patients who died in the BWH RoCI at 28-days, 12 (20%) did not have a measurement of mtDNA that was elevated on the initial blood draw. Of these 12 patients, advanced stage cancer (mostly metastatic disease) was present in 9 patients (including non-small cell cancer of the lung, colon cancer, breast cancer, pancreatic cancer (2), Hodgkin’s disease, cholangiocarcinoma, renal cancer, and acute myelogenous leukemia) while 3 patients had other diagnoses including anoxic brain death after status asthmaticus, atrial fibrillation, aspiration pneumonitis. Of note, 4 of these 12 patients had a repeat measurement of mtDNA at ICU day 7, and in all 4 of these patients mtDNA increased to a level > 3200 copies/μl.

**Text S6:**

Nuclear DNA level and 28-day mortality

Comparable to our findings with mtDNA levels, median nuclear DNA levels were higher in those who died within 28 days of MICU admission (median 479,409 copies/µl) than in those who did not (174,896 copies/µl) in the BWH RoCI cohort (P=8x10^-8^ for comparison). Despite the strong evidence for association between nuclear DNA level and 28-day mortality those with an mtDNA level >3200 copies/µl had an increase in their odds of dying within 28 days of MICU admission even in analyses adjusting for age, gender, race, APACHE II score, and nuclear DNA level (OR 5.1, 95% CI 2.3-11.0, P=4x10^-5^). In this combined analyses there was no additional association between nuclear DNA level (P=0.23) and 28-day mortality after accounting for an elevated mtDNA level.

**Text S7:**

MtDNA level and overall mortality in the BWH RoCI

There was no evidence that the effect of mtDNA level on the risk of death overall was attenuated in models adjusting for age, gender, race, and APACHE II score, sepsis status or ARDS status (a mtDNA level >3200 copies/μl was associated an increase in the risk of death, HR 2.7, 95% CI 1.7-4.1, *P* < 1x10^-4^).

**Text S8:**

MtDNA level and 28-day mortality in combined analyses

In combined analyses of both cohorts, patients with an mtDNA level >3200 copies/µl had an increase in their odds of dying within 28 days of MICU admission in both unadjusted and adjusted analyses (**Table S4**). The magnitude of this association was greater when analyses were restricted to MICU patients (**Table S4**). Similarly, in net reclassification analyses there was evidence that the inclusion of an mtDNA level of >3200 copies/µl resulted in a 43% improvement in net reclassification when added to a clinical model (including age, gender, racial and ethnic category, APACHE II score, and sepsis, see **Table S4**). While the inclusion of an mtDNA level of >3200 copies/µl resulted in a significant improvement in the net reclassification when added to a clinical model regardless of varying risk categories, the improvement in net reclassification was reduced when the number of risk categories was reduced (**Table S4**). Similar to our findings with association, measures of net reclassification were improved regardless of varying risk categories in analyses limited to MICU patients. Similar to our findings with net reclassification, in combined analyses there was evidence that an mtDNA level >3200 copies improved 28-day ICU mortality prediction (assessed by a comparison of c-statistics) when added to a model with clinical variables (the c-statistic was 0.79 for a model including age, gender, race, sepsis status, and APACHE II score, which improved to 0.81 with the inclusion of an elevated mtDNA level, P < 1x10^-4^ for comparison, see **Table S4**), with similar findings noted when analyses were limited to MICU patients (**Table S4**).

**Figure Legend**

**Figure S1:** Plot of the threshold cycle (Ct) against the input DNA concentration of the samples after the qPCR using bacterial DNA were shown. DNA isolated from *Escherichia coli* (Gram negative bacteria), *Enterococcus faecalis* (Gram positive bacteria) and *Clostridium difficile* (Anaerobic bacteria) were subject to qPCR analysis using primers for human mtDNA primers or bacterial 16S ribosomal RNA (Bacterial 16S). While bacterial 16s primers amplified DNA from each of our bacterial samples, human mtDNA primers were not able to amplify DNA from any bacterial sample.

**Table S1:** BLAST search for bacterial genome

1. Human mtDNA primer (Forward)

| **Hit_def** | **Hsp_evalue** |
| --- | --- |
| **Homo sapiens mitochondrion, complete genome** | **0.022** |
| Oceanithermus profundus DSM 14977 chromosome, complete genome | 1.31673 |
| Pseudomonas protegens CHA0, complete genome | 5.2029 |
| Streptomyces fulvissimus DSM 40593, complete genome | 5.2029 |
| Streptomyces sp. PAMC26508, complete genome | 5.2029 |
| Streptomyces venezuelae ATCC 10712, complete genome | 5.2029 |
| Vibrio furnissii NCTC 11218 chromosome 1, complete sequence | 5.2029 |
| Streptomyces flavogriseus ATCC 33331 chromosome, complete genome | 5.2029 |
| Olsenella uli DSM 7084 chromosome, complete genome | 5.2029 |
| Geodermatophilus obscurus DSM 43160 chromosome, complete genome | 5.2029 |
| Streptomyces griseus subsp. griseus NBRC 13350 chromosome, complete genome | 5.2029 |
|  |  |

1. Human mtDNA (Reverse)

| **Hit_def** | **Hsp_evalue** |
| --- | --- |
| **Homo sapiens mitochondrion, complete genome** | **0.022** |
| Rhodanobacter sp. 2APBS1, complete genome | 1.31391 |
| Haliscomenobacter hydrossis DSM 1100 chromosome, complete genome | 1.31391 |
| Methylotenera mobilis JLW8 chromosome, complete genome | 1.31391 |
| Burkholderia sp. RPE64 DNA, chromosome 1, complete genome | 5.19176 |
| Terriglobus roseus DSM 18391 chromosome, complete genome | 5.19176 |
| Desulfitobacterium hafniense Y51 chromosome, complete genome | 5.19176 |
| Enterobacter sp. R4-368, complete genome | 20.5146 |
| Francisella tularensis subsp. holarctica F92 chromosome, complete genome | 20.5146 |
| Francisella tularensis subsp. holarctica FSC200 chromosome, complete genome | 20.5146 |
| Francisella tularensis subsp. tularensis NE061598 chromosome, complete genome | 20.5146 |

1. Human cytochrome B (Forward)

| **Hit_def** | **Hsp_evalue** |
| --- | --- |
| **Homo sapiens mitochondrion, complete genome** | **0.087** |
| Chitinophaga pinensis DSM 2588 chromosome, complete genome | 0.0880764 |
| Chloroflexus sp. Y-400-fl chromosome, complete genome | 1.37517 |
| Pseudomonas putida GB-1 chromosome, complete genome | 1.37517 |
| Chloroflexus aurantiacus J-10-fl chromosome, complete genome | 1.37517 |
| Bacillus sp. 1NLA3E, complete genome | 5.4338 |
| Ignavibacterium album JCM 16511 chromosome, complete genome | 5.4338 |
| Zobellia galactanivorans chromosome, complete genome | 5.4338 |
| Runella slithyformis DSM 19594 chromosome, complete genome | 5.4338 |
| Shewanella woodyi ATCC 51908 chromosome, complete genome | 5.4338 |
| Enterobacter sp. 638, complete genome | 5.4338 |

1. Human cytochorome B (Reverse)

| **Hit_def** | **Hsp_evalue** |
| --- | --- |
| **Homo sapiens mitochondrion, complete genome** | **0.022** |
| Pseudomonas stutzeri ATCC 17588 = LMG 11199 chromosome, complete genome | 5.42266 |
| Methylotenera versatilis 301 chromosome, complete genome | 5.42266 |
| Rhodococcus opacus B4, complete genome | 5.42266 |
| Chlorobium phaeovibrioides DSM 265 chromosome, complete genome | 5.42266 |
| Rhodococcus jostii RHA1 chromosome, complete genome | 5.42266 |
| Syntrophus aciditrophicus SB chromosome, complete genome | 5.42266 |
| Acinetobacter sp. ADP1 chromosome, complete genome | 5.42266 |
| Listeria monocytogenes strain J1926, complete genome | 21.4269 |
| Listeria monocytogenes strain J1776, complete genome | 21.4269 |
| Listeria monocytogenes strain R2-502, complete genome | 21.4269 |

**Table S2:** BLAST search for human genome

1. Human mtDNA (Forward)

| **Hit_def** | **Hsp_evalue** |
| --- | --- |
| **Homo sapiens mitochondrion, complete genome** | **0.021929** |
| Homo sapiens chromosome 22 genomic scaffold, alternate assembly CHM1_1.0 | 1.35289 |
| Homo sapiens chromosome 22 genomic scaffold, alternate assembly CHM1_1.0 | 1.35289 |
| Homo sapiens chromosome 22 genomic scaffold, alternate assembly HuRef SCAF_1103279188372 | 1.35289 |
| Homo sapiens chromosome 22 genomic scaffold, alternate assembly HuRef SCAF_1103279188372 | 1.35289 |
| Homo sapiens chromosome 22 genomic contig, GRCh37.p10 Primary Assembly | 1.35289 |
| Homo sapiens chromosome 22 genomic contig, GRCh37.p10 Primary Assembly | 1.35289 |
| Homo sapiens chromosome 5 genomic scaffold, alternate assembly HuRef SCAF_1103279188146 | 5.34577 |
| Homo sapiens chromosome Y genomic scaffold, alternate assembly HuRef SCAF_1103279188422 | 5.34577 |
| Homo sapiens chromosome Y genomic contig, GRCh37.p10 Primary Assembly | 5.34577 |
| Homo sapiens chromosome 2 genomic scaffold, alternate assembly CHM1_1.0 | 5.34577 |

1. Human mtDNA (Reverse)

| **Hit_def** | **Hsp_evalue** |
| --- | --- |
| **Homo sapiens mitochondrion, complete genome** | **0.021929** |
| Homo sapiens chromosome 2 genomic scaffold, alternate assembly CHM1_1.0 | 1.35289 |
| Homo sapiens chromosome 11 genomic scaffold, alternate assembly CHM1_1.0 | 1.35289 |
| Homo sapiens chromosome 11 genomic scaffold, alternate assembly CHM1_1.0 | 1.35289 |
| Homo sapiens chromosome 11 genomic scaffold, alternate assembly CHM1_1.0 | 1.35289 |
| Homo sapiens chromosome 17 genomic scaffold, alternate assembly CHM1_1.0 | 1.35289 |
| Homo sapiens chromosome X genomic scaffold, alternate assembly CHM1_1.0 | 1.35289 |
| Homo sapiens chromosome X genomic scaffold, alternate assembly HuRef SCAF_1103279188413 | 1.35289 |
| Homo sapiens chromosome 17 genomic scaffold, alternate assembly HuRef SCAF_1103279188370 | 1.35289 |
| Homo sapiens chromosome 11 genomic scaffold, alternate assembly HuRef SCAF_1103279188268 | 1.35289 |
| Homo sapiens chromosome 2 genomic scaffold, alternate assembly HuRef SCAF_1103279188159 | 1.35289 |

1. Human cytochrome B (Forward)

| **Hit_def** | **Hsp_evalue** |
| --- | --- |
| Homo sapiens chromosome 5 genomic scaffold, alternate assembly CHM1_1.0 | 0.021929 |
| Homo sapiens chromosome 5 genomic contig, GRCh37.p10 Primary Assembly | 0.021929 |
| **Homo sapiens mitochondrion, complete genome** | **0.0866495** |
| Homo sapiens chromosome 5 genomic scaffold, alternate assembly CHM1_1.0 | 5.34577 |
| Homo sapiens chromosome 5 genomic contig, GRCh37.p10 Primary Assembly | 5.34577 |
| Homo sapiens chromosome 5 genomic scaffold, alternate assembly CHM1_1.0 | 21.1231 |
| Homo sapiens chromosome 5 genomic contig, GRCh37.p10 Primary Assembly | 21.1231 |
| Homo sapiens chromosome 2 genomic scaffold, alternate assembly CHM1_1.0 | 21.1231 |
| Homo sapiens chromosome 2 genomic scaffold, alternate assembly CHM1_1.0 | 21.1231 |
| Homo sapiens chromosome 16 genomic scaffold, alternate assembly CHM1_1.0 | 21.1231 |
| Homo sapiens chromosome 16 genomic scaffold, alternate assembly HuRef SCAF_1103279188144 | 21.1231 |

1. Human cytochrome B (Reverse)

| **Hit_def** | **Hsp_evalue** |
| --- | --- |
| **Homo sapiens mitochondrion, complete genome** | **0.021929** |
| Homo sapiens chromosome 5 genomic scaffold, alternate assembly CHM1_1.0 | 0.021929 |
| Homo sapiens chromosome 5 genomic scaffold, alternate assembly HuRef SCAF_1103279188412 | 0.021929 |
| Homo sapiens chromosome 5 genomic contig, GRCh37.p10 Primary Assembly | 0.021929 |
| Homo sapiens chromosome 5 genomic scaffold, alternate assembly CHM1_1.0 | 5.34577 |
| Homo sapiens chromosome 6 genomic scaffold, alternate assembly CHM1_1.0 | 5.34577 |
| Homo sapiens chromosome 8 genomic scaffold, alternate assembly CHM1_1.0 | 5.34577 |
| Homo sapiens chromosome 15 genomic scaffold, alternate assembly CHM1_1.0 | 5.34577 |
| Homo sapiens chromosome 16 genomic scaffold, alternate assembly CHM1_1.0 | 5.34577 |
| Homo sapiens chromosome 16 genomic scaffold, alternate assembly HuRef SCAF_1103279188406:1-4552159 | 5.34577 |
| Homo sapiens chromosome 8 genomic scaffold, alternate assembly HuRef SCAF_1103279188282 | 5.34577 |

**Table S3**: Multivariate Logistic Regression Analysis of Association of the Odds for 28-day Mortality in the BWH RoCI

|  | Multivariate Logistic Regression Model | |
| --- | --- | --- |
| Variables in model | Odds Ratio (95% Confidence Interval) | *P* value |
| MtDNA elevated level  (reference <3200 copies/μl) | 9.48 (3.51-25.61) | 3 x 10^-5^ |
| Age  (per each 1 year increase) | 1.01 (0.98-1.04) | 0.59 |
| Sex  (reference males) | 0.51 (0.21-1.26) | 0.15 |
| Race  (reference whites) | 0.28 (0.07-1.18) compared to AA  0.13 (0.01-1.69) compared to Hispanic  0.39 (0.03-5.81) compared to Asians | 0.91  0.35  0.74 |
| Apache II score  (per each 1 unit increase) | 1.10 (1.02-1.18) | 0.01 |
| Sepsis | 0.15 (0.05-0.48) | 0.001 |
| ARDS | 1.18 (0.35-3.98) | 0.79 |
| Vasopressor use | 2.26 (0.90-5.69) | 0.08 |
| Cancer | 4.55 (1.75-11.81) | 0.002 |
| Acute Kidney Injury^a^ | 0.82 (0.30-2.25) | 0.70 |
| Need for Mechanical Ventilation | 1.60 (0.54-4.73) | 0.39 |
| Heart Failure^b^ | 0.22 (0.04-1.12) | 0.08 |
| Liver Failure^b^ | 3.23 (0.85-12.32) | 0.09 |

Abbreviations: BWH RoCI, Brigham and Women’s Hospital Registry of Critical Illness; AA, African-American; APACHE II, The Acute Physiology and Chronic Health Evaluation II score; ARDS, Adult Respiratory Distress Syndrome.

**^a^** Acute Kidney Injury is defined by 0.5 mg/dl increase in serum creatinine.

**^b^** Based on initial admitting physician report.

**Table S4:** Combined analyses of an Elevated MtDNA Levelsa and 28-day MICU Mortality

| Association | | | Prediction | | | | | |
| --- | --- | --- | --- | --- | --- | --- | --- | --- |
| All Patients  BWH RoCI and ME ARDS combined  (n=443) | | All MICU Patients^b^  BWH RoCI and  ME ARDS combined  (n=326) | All Patients  BWH RoCI and ME ARDS combined  (n=443) | | | | All MICU Patients^b^  BWH RoCI and  ME ARDS combined†  (n=326) | |
|  | OR (95% CI),  P value§ | OR (95% CI),  P value§ |  | Risk Categories | NRI, SE,  P value^c^ | c-statistic  or c-statistic comparison,  p value | NRI, SE,  P value^c^ | c-statistic  or c-statistic comparison,  p value |
| Elevated MtDNA^a^ | 4.8 (2.9-8.2),  4 x 10^-9^ | 6.0 (3.3-11.0),  5 x 10^-9^ | Elevated MtDNA^a^ |  |  | 0.68 |  | 0.70 |
| Elevated MtDNA^a^ + Clinical Model^d^ | 3.7 (2.1-6.6),  6 x 10^-6^ | 5.0 (2.6-9.7),  1 x 10^-6^ | Elevated MtDNA^a^+ Clinical Model^d^ | Increments of 10% from 0%-100%^e^ | 43%, (10%), <0.0001 | 0.79 to 0.81, <0.0001 | 58%, (11%), <0.0001 | 0.77 to 0.81, <0.0001 |
|  |  |  | Elevated MtDNA^a^ + Clinical Model^d^ | Increments of 20% from 0%-100%^f^ | 18% (8%) 0.02 |  | 27% (9%) 0.003 |  |
|  |  |  | Elevated MtDNA^a^ + Clinical Model^d^ | Increments of 33%  From 0-100%^g^ | 14% (6%) 0.01 |  | 23% (7%)  9 x 10^-4^ |  |
|  |  |  | Elevated MtDNA^a^ + Clinical Model^d^ | Doubling increments  From 0-100%^h^ | 30% (8%)  2 x 10^-4^ |  | 40% (9%)  <0.0001 |  |

Abbreviations: BWH RoCI, Brigham and Women’s Hospital Registry of Critical Illness; ME ARDS, The Molecular Epidemiology of Adult Respiratory Distress Syndrome; OR (95% CI), Odds Ratio (95% Confidence Interval).

**^a^** Elevated MtDNA level represents NADH dehydrogenase 1 DNA copy number of >3200 copies/µl plasma.

**^b^** MICU patients represents Medical ICU patients including both medical ICU and cardiac ICU patients.

**^c^** NRI, SE, p value: Net reclassification improvement, standard error, p value (using risk categories in increments as designated). In all cases the net reclassification improvement refers to the addition of the noted biomarker to the clinical model (including age, gender, and racial and ethnic category, APACHE II score, and sepsis) and additional biomarkers where indicated

**^d^** Clinical Model includes age, gender, and racial and ethnic category, APACHE II score, and sepsis.

**^e^** Risk category increments include <10%, 10-20%, 20-30%, 30-40%, 40-50%, 50-60%, 60-70%, 70-80%, 80-90%, >90%.

**^f^**  Risk category increments include <20%, 20-40%, 40-60%, 60-80%, >80%.

**^g^** Risk category increments include <33%, 33-67%, >67%.

**^h^** Risk category increments include <5%, 5-10%, 10-20%, 20-40%, 40-80%, >80%.

**References for Supporting Information:**

1. Levy MM, Fink MP, Marshall JC, Abraham E, Angus D, et al. (2003) 2001 SCCM/ESICM/ACCP/ATS/SIS International Sepsis Definitions Conference. Crit Care Med 31: 1250-1256.

2. Bernard GR, Artigas A, Brigham KL, Carlet J, Falke K, et al. (1994) The American-European Consensus Conference on ARDS. Definitions, mechanisms, relevant outcomes, and clinical trial coordination. Am J Respir Crit Care Med 149: 818-824.

3. Zhang Q, Raoof M, Chen Y, Sumi Y, Sursal T, et al. (2010) Circulating mitochondrial DAMPs cause inflammatory responses to injury. Nature 464: 104-107.

4. Sursal T, Stearns-Kurosawa DJ, Itagaki K, Oh SY, Sun S, et al. (2013) Plasma bacterial and mitochondrial DNA distinguish bacterial sepsis from sterile systemic inflammatory response syndrome and quantify inflammatory tissue injury in nonhuman primates. Shock 39: 55-62.
