# Supplementary figures and images for: Circulating Mitochondrial DNA in Patients in the ICU as a Marker of Mortality: Derivation and Validation
Source: PLoS Med. 2013 Dec 31;10(12):e1001577. doi: 10.1371/journal.pmed.1001577 (PMC3876981; doi:10.1371/journal.pmed.1001577)

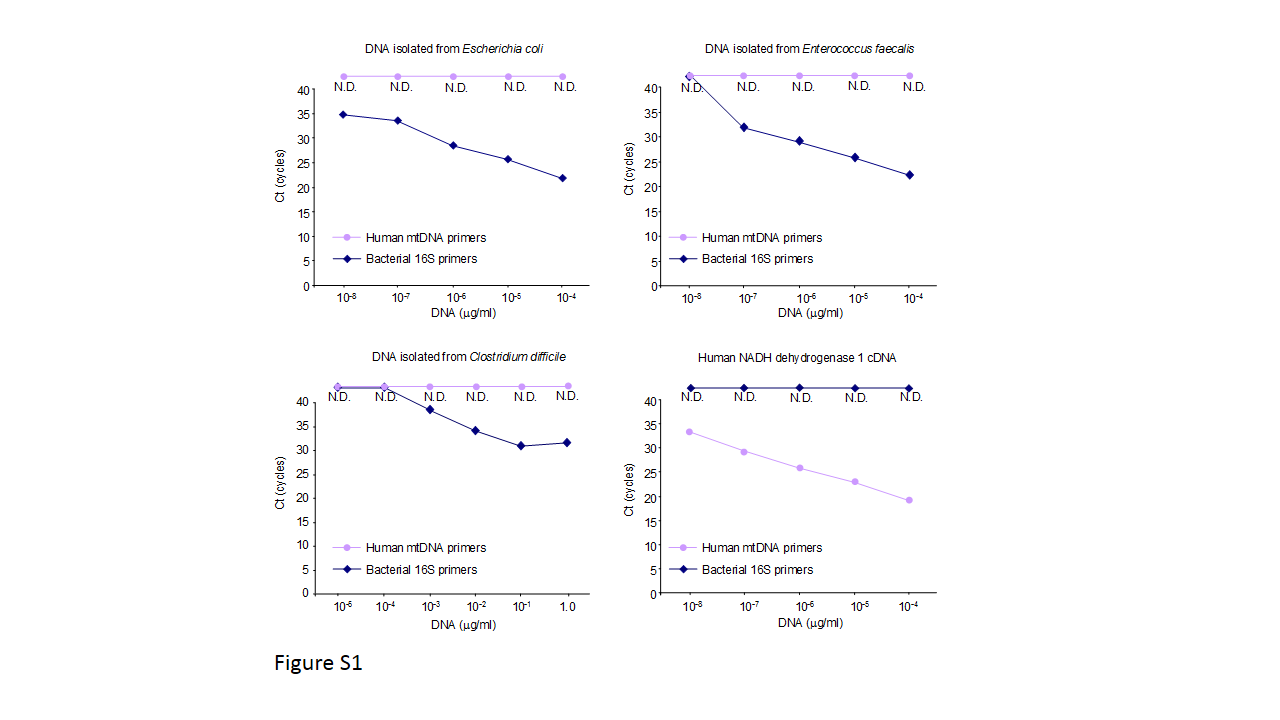

Supplement: Figure S1 — Plot of the threshold cycle (Ct) against the input DNA concentration of the samples after the qPCR using bacterial DNA. DNA isolated from Escherichia coli (Gram negative bacteria), Enterococcus faecalis (Gram positive bacteria), and Clostridium difficile (anaerobic bacteria) were subject to qPCR analysis using primers for human mtDNA primers or bacterial 16S ribosomal RNA (bacterial 16S). While bacterial 16S primers amplified DNA from each of our bacterial samples, human mtDNA primers were not able to amplify DNA from any bacterial sample. (TIF) [file pmed.1001577.s002.tif]
